# Evaluating the Resistance of *Eimeria* Spp. Field Isolates to Anticoccidial Drugs Using Three Different Indices

**Published:** 2013

**Authors:** F Arabkhazaeli, M Modrisanei, S Nabian, B Mansoori, A Madani

**Affiliations:** 1 Department of Pathobiology, Faculty of Veterinary Medicine, University of Tehran, Iran; 2 Department of Animal and Poultry Health and Nutrition, Faculty of Veterinary Medicine, University of Tehran, Iran; 3 Department of Clinical Sciences, Faculty of Veterinary Medicine, University of Tehran, Iran

**Keywords:** *Eimeria*, Drug resistance, Global Index, Anticoccidial Sensitivity Test, Optimum Anticoccidial Activity

## Abstract

**Background:**

In this study, the presence of resistance to diclazuril, amprolium+ethopabate and salinomycin, representing some of the commonest anticoccidials in Iran's poultry industry, against three mixed *Eimeria* field isolates were investigated.

**Methods:**

Three *Eimeria* field isolates, collected from typical broiler farms in Iran, were propagated once, inoculated to 480 broilers, comprising 30 chicks in each treatment. The non-medicated or medicated diets containing one of the above mentioned anticoccidials were provided *ad-lib*. Drug efficacy was determined using the Global index (GI), Anticoccidial Sensitivity Test (AST) and Optimum Anticoccidial Activity (OAA).

**Results:**

None of the field isolates were fully sensitive to the selected anticoccidials. All isolates showed reduced sensitivity/partial resistance to salinomycin. Resistance to amprolium+ethopabate was evident and partial to complete resistance was recorded for diclazuril.

**Conclusion:**

Limited efficacy of the selected anticoccidials is obvious. Considering the cost of continuous use of anticoccidials in the field, altering the prevention strategy and rotation of the anticoccidials with better efficacy, would prevent further economic losses induced by coccidiosis.

## Introduction

Amongst various parasitic infections, coccidiosis caused by obligate intracellular protozoan parasite of the genus *Eimeria*, is a major parasitic disease within the intensively reared poultry industry. *Eimeria* spp. are highly host-specific, with six species having economic impact on chicken (1). Coccidiosis is considered as one of the most significant diseases of poultry and costs the world's commercial chicken producers at least 1.5 billion US$ every year (2). The disease has caused great economic losses in the poultry industry of Iran like the other parts of the world (3). In broilers, coccidiosis control is not limited to the prevention of clinical disease and mortality, since mild and subclinical infections are also important as even minor intestinal lesions can interfere with growth and feed efficiency and therefore profitability. In addition to management measurements (litter condition, flock density); the disease has largely been controlled by directly adding anticoccidial drugs to the feed (4).

Following the introduction of most anticoccidial agents, drug resistant strains have been isolated from the field (2). The public concern of chemical residues in meat and pollution of the environment has led to stricter regulations against the use of coccidiostats in food in Europe (5–7); besides the increasing development of drug-resistant coccidian species has stimulated searches for alternative control methods such as applying a live vaccine early in life or development of new drugs (8).

The intensive use of anticoccidial drugs has led to the development of resistance (9). Different indices using different variables and interpretation of recorded variables to evaluate anticoccidial efficacy against *Eimeria* species were defined by scientists.

This study has been conducted to investigate the probability of resistance to some of the most commonly used anticoccidials in three *Eimeria* field isolates in Iran and to evaluate three usual anticoccidial efficacy indices used in the literature for measuring resistance.

## Materials and Methods

### Sample collection and Parasitology

Sample collection, parasite propagation and oocyst detection was performed from March to September 2010, similar to those previously described (10–12). Samples 1 and 2 were isolated from litters of two commercial broiler houses from two separate villages located in Mazandaran Province, north of Iran and sample 3 was isolated from broiler intestines originated from Hamadan Province, west of Iran.

### Animals

Four hundred and eighty male one-day-old Ross broiler chicks were randomly assigned to 16 dietary treatments containing 30 chicks, each comprised of three replicates of 10 and kept in battery cages under coccidia-free condition. The dietary treatments are summarized in Table 1. The chickens were fed a diet based on corn and soybean meal, which has been formulated to meet or exceed all required nutrients for the birds (13), and food and water were provided *ad-libitum* throughout the experimental period. No vaccine was used during the test period. On the beginning of the experiment (day 0 = 14th day of age), the birds were leg tagged so that individual data can be recorded.

**Table 1:** Anticoccidials and infective dose inoculated to each of the treatment groups

Treatment	Isolate*	Inoculated infective dose	Anticoccidials
1	1	300000 oocyst/bird	Salinomycin @ 500 ppm
2	2	300000 oocyst/bird	Salinomycin @ 500 ppm
3	3	250000 oocyst/bird	Salinomycin @ 500 ppm
4	1	300000 oocyst/bird	Amprolium+Ethopabate @ 500 ppm
5	2	300000 oocyst/bird	Amprolium+Ethopabate @ 500 ppm
6	3	250000 oocyst/bird	Amprolium+Ethopabate @ 500 ppm
7	1	300000 oocyst/bird	Diclazuril @ 200 ppm
8	2	300000 oocyst/bird	Diclazuril @ 200 ppm
9	3	250000 oocyst/bird	Diclazuril @ 200 ppm
10	1	300000 oocyst/bird	Non-medicated
11	2	300000 oocyst/bird	Non-medicated
12	3	250000 oocyst/bird	Non-medicated
13	Non-Infected	-	Non-medicated
14	Non-Infected	-	Salinomycin
15	Non-Infected	-	Amprolium+Ethopabate
16	Non-Infected	-	Diclazuril

### Purification and standardization of inoculum doses for experimental infection

Based on observations during propagation and isolate composition, the proper doses for challenge were estimated, so that maximum lesions but minimal mortality is caused (12) (Table 1). According to shape index, the different species of *Eimeria* present in the three inoculums were as follow: isolate 1) 12% *E. acervulina*, 16% *E. runetti*, 44% *E. maxima*, 12% *E. mitis*, 12% *E. tenella*, 4% *E. necatrix*; isolate 2) 24% *E. acervulina*, 6% *E. brunetti*, 34% *E. maxima*, 16% *E. mitis*, 18%*, E. tenella*, 2%, *E.necatrix* and isolate 3) 40% *E. acervulina*, 15% *E. brunetti*, 25% *E. maxima, 8*%, *E. mitis*, 6%*,E. tenella*, and 6% *E.necatrix*.

### Challenge study

Kimiamycin^®^ 12 (salinomycin 12%, SAL) (500 ppm) (Kimiafaam Group, Iran), Ethoamprox^®^ (amprolium 20%+ethopabate 6.1%, AMP) (500 ppm) (JamedatAfagh Pharmaceutical Company, Iran) and Clinacox^®^ (diclazuril l0.5%, DIC) (200 ppm) (Kimiafaam Group, Iran) were included in the feed at 12 days of age (48 h before inoculation of the infective dose) and continued up to 7 days post-inoculation. The infectious dose was given orally on 14th day of age. All birds were weighed individually on the day of infection and on 21st day of age (7 days post inoculation). Data regarding weight gain (WG), feed intake (FI), lesion score (LS), oocysts index (OI) and mortality were recorded. Feed conversion ratio (FCR) was calculated (11, 12, 14).

### Evaluation of resistance

For each isolate, the observations were combined and three different indices were calculated to evaluate the anticoccidial efficacy as follows:

### Global index

The formula, first developed by Stephan et al. (1997), includeing weight gain, the feed conversion for the treatment group and the negative control (NNC), oocyst index and the lesion score for the treatment group and the infected/non-medicated control (INC) (2 & 15). An oocyst index of 0 to 5 was determined by examination of scrapings from each four segment of intestine for birds sacrificed for lesion score at 7^th^ day post- inoculation (12 & 14). The GI for each dietary treatment was calculated as percentage of the GI for the negative control group according to the following 5 categories:

≥90% GI_NNC_: Very good efficacy

80-89% GI_NNC_: Good efficacy

70-79% GI_NNC_: Limited efficacy

50-69% GI_NNC_: Partially resistant

<50% GI_NNC_: Resistant

### Optimum Anticoccidial Activity (OAA)

In this index, a growth and survival ratio (GSR) is used to calculate the percentage of Optimum Anticoccidial Activity for each treatment as follows:

Resistant if ≤50%, partially resistant if 51%-74% and sensitive if ≥75% (16).

### Anticoccidial Sensitivity Test (AST)

The anticoccidial sensitivity test is calculated based on the reduction of mean lesion score of the treatment group compared with the infected non-medicated group (INC). A reduction of 0 to 30%, 31-49% and at least 50% indicates resistance, reduced sensitivity/partial resistance and full sensitivity, respectively (17).

### Statistical analysis

All data were subjected to ANOVA and two way t-test to see whether the differences among groups are significant (SPSS 15.0 for Windows, SPSS, Chicago, IL). Tukey's multiple range tests was used to test the significance of different treatments at *P*≤0.05.

## Results

### Sensitivity to anticoccidials

The results of the battery cage trial are summarized in Table 2. In all treatments, WG of the treatment groups were significantly different with NNC (*P*≤0.05) and the highest WG was recorded for the NNC group. In salinomycin medicated groups, weight gain, FCR and LS were better than that of INC groups (*P*≤0.05). However, OI of infected-medicated birds challenged to isolates 1, 2 and 3 did not show significant difference with related INC groups. Amprolium+ethopabate could not improve WG and FCR of birds inoculated by isolates 1 and 2, comparing with the related INC group (*P*≤0.05), however the mentioned parameters and OI of birds challenged by isolate 3, LS of birds inoculated by isolate 1 and LS and OI of birds infected by isolate 2 were different from their corresponding INC group (*P*≤0.05). Considering the birds medicated with diclazuril, WG, FCR and LS of birds inoculated by isolate 3 showed significant differences with the analogous INC group (*P*≤0.05). No difference was observed for OI of birds inoculated by either of the isolates with their related INC groups (*P*≤0.05). Diclazuril could not increase the WG of birds challenged by isolates 1 and 2 in comparison with their related INC groups (*P*≤0.05).

**Table 2:** Evaluation of developing resistance in three chicken *Eimeria* field isolates in coccidian-infected broiler chicks

Experimental group		AWG (g)	FCR (g/g)	Lesion score	Oocyst index	%GI_NNC_	%OAA	AST			
								Ea	Em	Emi	Et
**Isolate 1**	Salinomycin	307.4^b^	1.7^C^	0.7^b^	1.3	LE	PR	S	PR	S	S
	Amprolium+ethopabate	252.7^C^	2.01^a^	0.9^b^	1.5	PR	R	PR	S	R	S
	Diclazuril	254.5^C^	1.9^b^	0.9^b^	1.6	PR	R	PR	PR	S	S
	INC	237.1^C^	2.03^a^	1.9^a^	1.4						
	NNC	348.5^a^	1.6^C^	-	-						
	S.E.M.	12.0	0.04	0.14	0.14						
**Isolate2**	Salinomycin	284.9^b^	1.77^bc^	0.8^b^	1.0^ab^	LE	PR	R	R	R	S
	Amprolium+ethopabate	213.7^C^	2.1^a^	0.9^b^	1.6^a^	R	R	R	R	PR	S
	Diclazuril	224.3^C^	1.9^ab^	1.0^b^	1.1^ab^	PR	R	R	R	R	S
	INC	205.6^C^	2.03^a^	1.5^a^	0.8^b^						
	NNC	348.5^a^	1.65^C^	-	-						
	S.E.M.	14.9	0.04	0.09	0.13						
**Isolate 3**	Salinomycin	249.6^b^	1.74^C^	1.5^bc^	1.5^ab^	PR	PR	R	PR	PR	PR
	Amprolium+ethopabate	196.9^C^	2.1^b^	2.1^ab^	1.2^b^	R	R	R	PR	R	R
	Diclazuril	258.1^b^	1.7^C^	1.2^C^	1.9^ab^	PR	PR	R	PR	R	S
	INC	145.3^d^	2.75^a^	2.3^a^	2.6^a^						
	NNC	348.5^a^	1.65^C^	-	-						
	S.E.M.	18.6	0.12	0.17	0.23						

a-c Means sharing the same superscripts within each section do not differ (P ≤ 0.05).

AWG: Average weight gain: FCR: Feed conversion ratio; INC: Infected Non-medicated Control group; NNC: Non- Infected Non- medicated Control group;%GI_NNC_=%WG_NNC_-[(F_M_-F_NNC_)×10]-(OI_M_-OI_INC_)-[(LS_M_-LS_INC_)×2)]-(%mortality/2) where GI is Global Index, WG is weight gain, F is FCR, OI is oocyst index, LS is lesion score, M is medicated group;%OAA=[average GSR of medicated group-average GSR of INC]/average GSR of NNC-average GSR of INC]×100 where OAA is Optimum Anticoccidial Activity, GSR is Growth and Survival Ratio calculated as the cage weight at trial termination+the weight of any dead bird/the cage weight when infected; AST= 100%-(mean lesion score of medicated group-mean lesion score of INC×100%) wher AST is Anticoccidial Sensitivity Test; *Ea: Eimeria acervulina; Em: E. maxima; Emi: E. mitis; Et: E. tenella*; LE: Limited Efficacy; PR: Partially Resistant; RS: Reduced Sensitivity; R: Resistant; S: Sensitive;

### GI

Limited efficacy of salinomycin and partial resistance to amprolium+ethopabate and diclazuril were recorded for isolate 1 based on GI. For isolate 2 limited efficacy of salinomycin, resistance to amprolium+ethopabate and partial resistance to diclazuril were proved. Isolate 3 was partially resistant to salinomycin and diclazuril and resistant to amprolium+ethopabate. Isolate 1 was partially resistant to amprolium+ethopabate whereas isolates 2 and 3 were categorized resistant. Partial resistance was recorded for all of the isolates to diclazuril (Table 2).

### OAA

According to this index, the three isolates were partially resistant to salinomycin and resistant to amprolium+ethopabate. Isolates 1 and 2 were resistant and isolate 3 was partially resistant to diclazuril (Table 2).

### AST

As is shown by AST, in isolate 1 and 2, cecum dwelling species were obviously sensitive to the anticoccidial drugs and for species inhabiting upper, middle and lower intestines reduced sensitivity to complete resistance could be estimated. For isolate 3, resistance was more frequent and only *E. tenella* was sensitive for diclazuril (Table 2).

## Discussion

The intensive use of anticoccidial drugs has led to the development of resistance which can be detected by means of different indices and criteria (18). In this study, resistance to some of the most commonly used anticoccidials against three *Eimeria* field isolates in Iran were investigated and three usual anticoccidial efficacy indices introduced in the literature (GI, OAA and AST) were used and compared.

The biopathologic characteristics of selected isolates including the species present in each isolate and other characteristics of the three field samples were previously described (12). Based on the results of this study, the reduction of WG, alteration of FI and subsequent alteration of FCR, in comparison to negative control, highlights the economic importance of the disease. Results of the sensitivity tests indicated that none of the field isolates was fully sensitive to the selected anticoccidials. All isolates showed reduced sensitivity/partial resistance to salinomycin. Resistance to amprolium+ethopabate was evident and partial to complete resistance was recorded for diclazuril.

To evaluate the degree of efficacy of anticoccidial compounds, a single parameter like lesion score (17, 19, 20), oocysts production (4, 21), or a combination of parameters including weight gain and oocysts production (22, 23), feed intake, feed conversion and lesion scores (24), mortality, weight gain, lesion and fecal scores (18, 25) are being used. Some indices are also introduced based on some of the above-mentioned parameters (15, 16, 26–29). Bird performance, particularly weight gain is widely used for assessing anticoccidial efficiency. However, it has to be considered that ionophores have a growth promoting effect per se. There is some debate on the variables such as lesion score and oocyst count (18, 30). Oocyst production is under influence of several factors such as reproduction potential of the various species, crowding, host defense and nutrition, competition with other species (31). Besides, different studies have proven that oocysts production does not necessarily correlate with weight gain, lesion score and even mortality (3, 15). Lesion score, as one of the commonest methods in assessing the condition of the intestine in coccidial infection, has deficiencies in evaluating anticoccidial efficacy due to its subjective nature, dependence on individual performing, lack of linear correlation with number of inoculated oocysts and weight gain (15, 18, 32).

As stated in the result section, assessment of drug efficacy based on different single parameters may lead to variable results. However based on the obtained results, both the GI and OAA give similar results and the dissimilarity is due to the narrower classification of the GI. Although the Global Index includes several parameters with coefficients corresponding to their profitability; weight gain as the most economically important parameter, with the largest weighting, feed conversion, oocyst excretion, intestinal lesion and percentage of mortality but considering the ease of weighting the birds in comparison to labor consuming task of recording lesion score and oocysts index, and because weight is the main factor profitability in production industry, it can be advised to use OAA as a valuable index in evaluating resistance till a more accurate method for resistance detection, such as molecular based methods would be developed.

## Conclusion

There were variations between various isolates in response to a similar anticoccidial drug but the best overall control was achieved by salinomycin followed by diclazuril and amprolium+ethopabate. There was no complete sensitivity detected for any of mixed species, although sensitive species could be classified according to the obtained results by AST index. From the obtained results, limited efficacy of the selected anticoccidials is obvious. Considering the cost of continuous use of anticoccidials in the field and noting that clinical or subclinical coccidiosis may develop in spite of adding anticoccidials to the feed, altering the prevention strategy and rotation of the anticoccidials with better efficacy, would prevent further economic losses induced by coccidiosis. From the literature and based on the present study, use of an index is preferred to a single parameter and because of undemanding task of measuring group weight in OAA, this index can be proposed as the single means for evaluating drug resistance so that researches based on a common method, can be compared with each other.
